# Autosomal Recessive Spastic Paraplegia and Psychomotor Retardation With or Without Seizures: A Case Report From Saudi Arabia

**DOI:** 10.7759/cureus.60642

**Published:** 2024-05-20

**Authors:** Hamd Alzaidan, Bashaer Alluhaybi, Naif A Albulayhid, Khalid H Al-jabr, Faihan T Alotaibi, Assem Alqahtani

**Affiliations:** 1 Department of Medical Genomics, Center for Genomic Medicine, King Faisal Specialist Hospital and Research Center, Riyadh, SAU; 2 College of Medicine, Prince Sattam Bin Abdulaziz University, Al-Kharj, SAU

**Keywords:** genetic mutation, seizure, spastic paraplegia, neurodevelopmental syndrome, hace1

## Abstract

Spastic paraplegia and psychomotor retardation with or without seizures (SPPRS) is a rare neurodevelopmental disorder associated with autosomal recessive mutations in the HACE1 gene. This case report presents the clinical features and genetic analysis of an 11-month-old girl and her sister with SPPRS, making it the third reported case in the Middle East and the second in Saudi Arabia. The patient exhibited hypotonia, global developmental delay, speech delay, swallowing difficulties, and recurrent respiratory infections. A homozygous pathogenic variant in the HACE1 gene (p.R664*) was identified through genetic analysis, confirming the diagnosis of SPPRS. This case report emphasizes the importance of considering variations in clinical presentation, especially in rare disorders where only a few cases are reported. Further research and case studies are needed to better understand the complete phenotypic spectrum of SPPRS and its complications.

## Introduction

Spastic paraplegia and psychomotor retardation with or without seizures (SPPRS) is a neurodevelopmental disorder described so far in 26 patients of 14 unrelated families associated with autosomal recessive mutations throughout the HACE1 gene [1]. In the majority of cases, the clinical findings are infantile-onset psychomotor developmental delay with severe intellectual disability and poor speech acquisition; these findings are typically associated with seizures, mostly myoclonic seizures. Muscle hypotonia may be observed at birth or within the first four months of life; in addition, slowly progressive lower limb spasticity that impairs gait, ocular abnormalities, and incontinence are commonly associated [2]. Certain patients also have brain abnormalities, such as cerebral atrophy, hypoplastic corpus callosum, delayed myelination, and reduced white matter content [3]. Consanguinity and possibly endogamy may be essential factors in the generational transmission of these harmful autosomal recessive mutations, as evidenced by the variety of loss-of-function mutations in the HACE1 gene linked to a familial neurodevelopmental disorder reported in independent reports [4]. This case report is the third case in the Middle East and the second in Saudi Arabia to document the involvement of the HACE1 gene in a neurodevelopmental disorder.

## Case presentation

**Figure 1:**
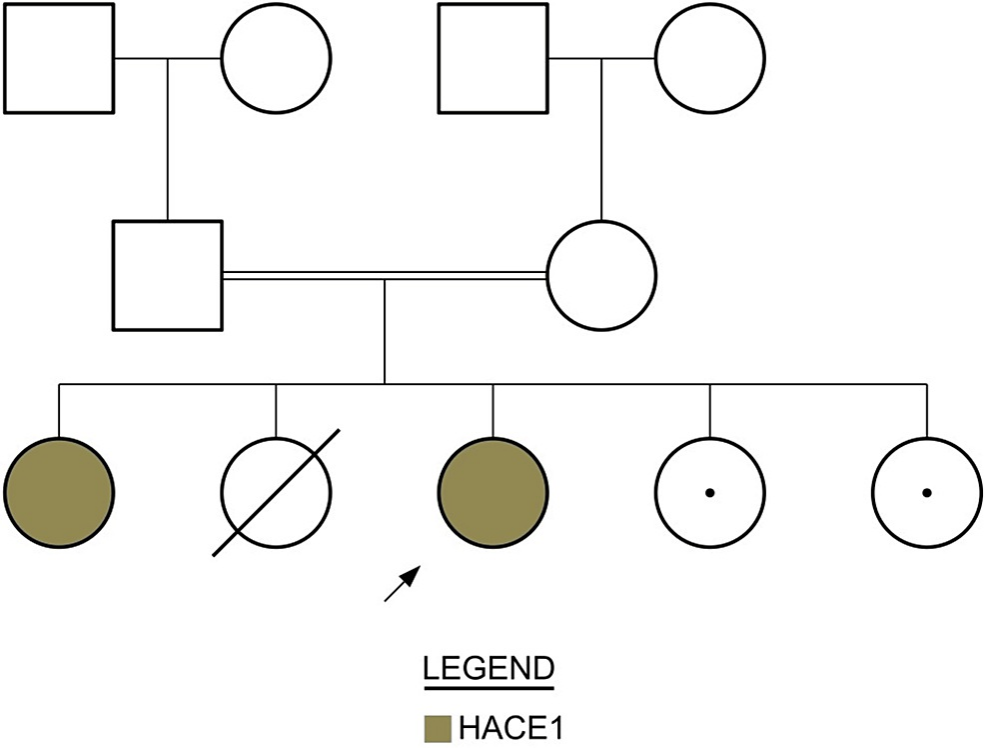
Simplified pedigree of the family with the affected individuals shaded

An 11-month-old girl was referred to King Faisal Specialist Hospital (KFSH) from Al-Qassim City with a history of hypotonia, developmental delay, swallowing difficulties, and repeated chest infections to rule out metabolic causes. She was a full-term product with an uneventful labor who was admitted to the neonatal intensive care unit (NICU) immediately after birth due to hypoglycemia, which was resolved after a week. She was healthy until she was admitted with severe pneumonia at the age of two months. During that one-month hospital stay, her parents observed that she was becoming hypotonic and less interactive. A brain magnetic resonance imaging (MRI) was performed in the local hospital and reported as normal. Following her discharge, she was readmitted again at the age of five months with symptoms of acute gastroenteritis and chest infections. The family noted occasional fever and a lack of sweat production; the patient had been on oral feeding since birth (bottle-feeding), with poor sucking skills (requiring four hours to consume her meal) and inconsistent choking. A nasogastric tube (NGT) was inserted at 10 months, with only a noted improvement in her weight gain. She was still having frequent chest infections. No history of seizures, decreased level of consciousness, diarrhea, or jaundice was reported. Her parents are relatives within the third degree of consanguinity. She has one sister, aged 10 years, with global developmental delay, hypotonia, and was diagnosed with cerebral palsy (CP); another sister who passed away at five months from an unknown cause; and two healthy sisters (Figure 1). She also has cousins from both the maternal and paternal sides diagnosed with CP. She has motor developmental delays and is unable to sit independently. On examination, she was below the third percentile with phenotypic changes like microcephaly, delayed tooth eruption, hemangioma involving the right eyelid and forehead, noticeable head lag, and severe hypotonia. She cannot sit on her own, and she cannot crawl as well. At 17 months, her metabolic workup, including tandem mass spectrometry (TMS), biotinidase activity, very long-chain fatty acid, and phytanic acid, were unremarkable. Her single nucleotide polymorphisms (SNP) array was negative. The urine test for creatine was normal as well. The patient's DNA was then extracted from a blood sample and analyzed by whole exon sequencing (WES), where all the genes encoding for proteins were aligned with a reference sequence, a pathogenic variant that can explain the phenotypic changes was shown, and a homozygous pathogenic variant in the HACE1 gene (p.R664*) was identified. Table 1 lists the pathogenic variants in disease genes related to the clinical phenotype. Her elder sister, who had the same phenotype, was positive for the same variant by target mutation analysis. Defects in HACE1 may cause spastic paraplegia and psychomotor retardation with or without seizures (SPPRS), an autosomal recessive complex neurodevelopmental disorder with onset in infancy. Affected children showed hypotonia, followed by severely impaired global developmental delay and significant motor disability. The patient was referred to the pediatric neurology clinic for a neurological assessment. On neurological examination, there is axial hypotonia and head lag, no appendicular hypotonia, and the tone in the upper limbs (UL) is normal bilaterally but slightly increased in the lower limb (LL).

**Table 1:** Results table showing the pathogenic variants in disease genes related to the clinical phenotype

Disease	Inheritance Pattern	Gene	Position	Isoform	Location	Nucleotide	Amino acid
Spastic paraplegia and psychomotor retardation with or without seizures {omim:616756}	AR	HACE1	CHR6:105219824	NM_020771.3	Exonic	C.1990C>T	p.R664*

The patient was then referred to the pediatric immunology department. An immunological workup showed her normal results, including acceptable CBC, immunoglobulin level, immunoglobulin G (IgG) antibodies to pneumococcal and tetanus vaccines, normal lymphocyte markers, and naive T-cell percentage.

After three months, the patient was transferred from the medical genetics clinic to the emergency department due to respiratory distress, high-grade fever, and desaturations; she was admitted to the pediatrics department as a case of aspiration pneumonia. A modified barium swallow study was ordered and showed oropharyngeal dysphagia. Subsequently, the patient was scheduled and had nasogastric tube insertion with fundoplication. Following that, the patient presented at the local hospital with the same symptoms, had a positive urine culture for Enterococcus faecium, developed pan-sensitive Pseudomonas aeruginosa one month prior, and received eight days of IV ceftazidime treatment.

An echocardiogram was done to rule out vegetation. It showed a moderate-sized secundum atrial septal defect with a left to right shunt and a mildly dilated right atrium with no evidence of vegetation. Then, the patient was admitted to the pediatric intensive care unit (PICU) for hypoxic respiratory failure secondary to presumed pneumonia, requiring a high-flow nasal cannula.

## Discussion

SPPRS is a rare neurodevelopment disorder with an autosomal recessive inheritance that occurs in infancy. A mutation in the HACE1 gene causes it, and it has different clinical presentations that make it hard to recognize on a clinical ground. Children with this condition will always exhibit global developmental delays.

The presented case report describes an 11-month-old girl with hypotonia and global developmental delay, as she is unable to sit independently and has speech delay, swallowing difficulties, and recurrent respiratory infections. The genetic analysis revealed a homozygous pathogenic variant in the HACE1 gene (p.R664*), confirming the diagnosis of SPPRS. All clinical features of previous studies are summarized in Table 2. Some of the clinical presentations of this patient, like hypotonia, developmental delay, and microcephaly, are consistent with previously reported SPPRS cases [1,7]. Oropharyngeal dysphagia that leads to subsequent recurrent respiratory infections was diagnosed in this patient using a modified barium swallow study and was not extensively discussed in the existing literature on SPPRS. She has a normal tone in upper limbs but slightly increased in lower limbs while other studies have reported similar findings or even both limbs exhibiting spasticity with greater involvement of the lower limbs, this highlights a notable distinction in the observed pattern of tone abnormalities [1,6]. The absence of ocular disorders, such as strabismus or retinal dystrophy, hearing loss, genital disorders like hypogonadism, or coarse facial features in our case is similar to findings in another study [4], in contrast to their presence in other studies. This emphasizes a notable contrast in the clinical presentation [2,6]. Other features observed in this patient, like delayed tooth eruption and hemangioma involving the right forehead and eyelid, have also not been reported in the literature on SPPRS. These unique findings suggest potential phenotypic variability within the SPPRS spectrum.

**Table 2:** An updated overview of HACE1 gene mutations The table includes information on various families from different countries, highlighting clinical features such as seizures, developmental delay, and skeletal abnormalities.

Clinical features	This study	Nagy V et al. (2019) [2]	Reuter MS et al. (2017) [5]		Hollstein R et al. (2015) [6]					Ugarteburu O et al. (2020) [3]	Hariharan N et al. (2018) [4]	Hollstein R et al. (2015) [6]			Nagy V et al. (2019) [2]		Kovalskaia VA et al. (2022) [1]	Akawi N et al. (2015) [7]						Issa MY et al. (2020) [8]			Alfares AA (2018) [9]
Mutation types	Family A CHR6 Exonic C.1990C>T p.R664*	Family A p.Q209*/p.Q209*	Family A c.402+5G>A/c.402+5G>A		FAMILY A p.R219*/p.R219*					Family A p.C80*/p.C80*	Family A p.W370*/p.W370*	Family B p.R748*/p.P674Ffs*5			Family B p.R332*/p.R332*		Family A ex 7 del/entire HACE1 deletion	Family A p.Q152*/p.Q618Vfs*3	Family B p.R269*/p.R269*	Family C p.C80*/p.C80*	Family D p.L832del/p.L832del			Family A c.2212-1G>A/c.2212-1G>A			Family A p.R664*/p.R664*
Origin	Saudi Arabia (consanguineous marriage)	Saudi Arabia (consanguineous marriage)	Syria (consanguineous marriage)		Pakistan (consanguineous marriage)					Pakistan (non-consanguineous marriage)	India (consanguineous marriage)	Germany (non-consanguineous marriage)			Turkey (consanguineous marriage)		Russian (non-consanguineous marriage)	n/a	n/a (consanguineous marriage)	n/a (consanguineous marriage)	n/a (consanguineous marriage)			n/a (consanguineous marriage)			n/a (consanguineous marriage)
Sex, Age	(Female) 11 months	(Female) 5 Years 4 months	(2 Females) n/a		(Male)18 years	(Male)15 years	(Male)22 years	(Male)19 years	(Female)3 years	(Male)10 years	(Male)11 years	(Female)10 years 11 months	(Male)7 years 8 months	(Male)7 years 8 months	(Female) 4 months	(Male) 6 months	(Male) 2.8-year	(Male) n/a	(Female) n/a	(Male) n/a	(2 Females, 1 Male) n/a			(2 Females + 1 Male) n/a			(Female)n/a
Hypotonia+developmental delay	Yes	Yes	Yes		Yes					Yes	Yes	Yes			Yes		Yes	Yes	Yes	Yes	Yes			Yes			n/a
Epilepsy, Seizures	None	None	n/a		Myoclonic and tonic-clonic epilepsy	Myoclonic and tonic-clonic	None	Tonic-clonic epilepsy	Myoclonic epilepsy	n/a	Myoclonic seizures in limbs	None	Myoclonic seizures, focal epilepsy	None	None		None	Yes	Yes	No	Yes			Seizures			n/a
Spasticity (Lower Limbs)	Yes	n/a	n/a		Bilateral spasticity					Paresis of the lower extremities with rigidity	Hypertonia and exaggerated deep tendon reflexes	Bilateral spasticity			n/a		Yes	Yes	n/a	n/a	Spasticity/ dystonia			Dystonia			Yes
Spasticity (Upper Limbs)	No	n/a	n/a		Normal	Dystonic	Normal	Normal	Increased tone	n/a	Hypertonia and exaggerated deep tendon reflexes	Normal	Dystonic posturing	Normal	n/a		Yes	n/a	n/a	n/a	n/a			n/a			n/a
CT/MRI brain	MRI of the brain, in this reported case, showed no abnormalities	Microcephaly, hypoplastic corpus callosum, brainstem abnormality, small sella with ectopic neurohypophysis, and mild ventriculomegaly	n/a		Cerebral underdevelopment and marked atrophy of frontal and temporal lobes.	No imaging	Generalised cerebral atrophy	Ventricular dilatation	Prominent generalized cerebral and brain stem atrophy, disproportion between grey and white matters.	Diffuse cortical atrophy and an arachnoid cyst in the right temporal lobe	Hypoplastic corpus callosum, lesions	Hypoplastic corpus callosum	Hypoplastic corpus callosum	Enlarged ventricles	n/a		Enlarged ventricles, cerebral atrophy	Hypoplastic corpus callosum, lesions						n/a			n/a
Other Features	Repeated chest infections, fevers, poor weight gain, microcephaly, delayed tooth eruption, hemangioma involving the right eyelid and forehead, and congenital heart defect	Mild facial dysmorphia, skeletal abnormalities, ulnar deviation of the wrists and small feet, bruxism, retinal dystrophy, and bilateral sensorineural loss	Recurrent infections		Wheelchair-bound, ocular abnormalities, skeletal defects, hypogenitalism, kyphoscoliosis, and overweight					3-methylglutaconic aciduria	Iris heterochromia, hypopigmented body hair, and skeletal defect (proximal femoral focal dysplasia)	Mild talipes equinovarus, lumbar lordosis			Small feet, enlarged head circumference, inverted and widely spaced nipples, facial dysmorphic features, Strabismus		Coarse face, mild hypertrichosis, and long eyelashes	Primary microcephaly	n/a	n/a	Primary microcephaly			Eye movement deficit, horizontal, Broad forehead, and hypertelorism			n/a

Furthermore, the identification of a congenital heart defect through an echocardiogram aligns with a previous study that has reported cardiac abnormalities in some individuals with SPPRS [7]. In contrast to other studies that reported neural imaging changes such as enlargement of ventricles, global brain atrophy, frontal/temporal lobe atrophy, and corpus callosum hypoplasia [2,6]. MRI of the brain in this reported case showed no abnormalities, highlighting a significant difference in the imaging findings.

## Conclusions

This case report provides insights into the clinical features and genetic analysis of SPPRS. It highlights the need for a multidisciplinary approach to diagnose and manage the condition while also emphasizing the importance of considering potential variations in clinical presentation and associated features. Further research and case studies are warranted to better understand the complete phenotypic spectrum of SPPRS and its associated complications.
